# The evolution of health services research in Austria: a bibliometric exploration of trends, themes, and collaborations

**DOI:** 10.3389/frhs.2025.1501035

**Published:** 2025-03-13

**Authors:** Kyung-Eun (Anna) Choi, Sebastian Fitzek

**Affiliations:** ^1^ Center for Health Services Research, Brandenburg Medical School, Neuruppin, Germany; ^2^ Health Services Research, Research Center Medical Image Analysis and Artificial Intelligence, Faculty of Medicine/Dentistry, Danube Private University, Krems_Stein, Austria

**Keywords:** bibliometric analysis, health services research, Austria, research trends, collaboration networks, digital health

## Abstract

**Background:**

Health services research (HSR) in Austria has expanded rapidly over the past two decades, reflecting the evolving need for a healthcare system that effectively addresses the broader challenges of an increasingly strained healthcare environment. Mapping the progression and focus areas of this research is essential for guiding policy-making and future studies.

**Objectives:**

This bibliometric study aims to chart the evolution of Austrian HSR between 2000 and 2024. By examining publication trends, thematic priorities, collaboration networks, and research impacts, the analysis provides evidence-based insights that inform healthcare strategies and highlight research gaps.

**Methods:**

A systematic literature search was conducted in PubMed, which targeted peer-reviewed articles published from 2000–July 31, 2024. In total, 81 articles met the inclusion criteria. Bibliometric methods, including coauthorship mapping, keyword co-occurrence analysis, and citation tracking, were used to identify core research themes, key authors, and institutional collaborations.

**Results:**

Annual publication outputs increased notably from 2019 to 2020, corresponding to the heightened focus on healthcare during the COVID-19 pandemic. The major themes included mental health, patient care, public health, and disease management, with a growing interest in telemedicine and digital solutions. The Medical University of Vienna led publication activity, and strong international ties were evident, particularly with institutions in the UK and Germany. Citation analyses revealed varied research impacts, with some highly cited studies influencing policy debates and clinical practices.

**Conclusions:**

Austrian HSR has a dynamic trajectory, reflecting evolving national priorities and global healthcare challenges. Continued efforts are needed to address gaps involving underserved populations, integrate digital health technologies, and enhance economic evaluations of primary care reforms. Furthermore, better standardization in the reporting of funding sources and conflicts of interest is recommended to strengthen methodological rigor and public trust. By fostering collaboration, transparency, and comprehensive evaluations, HSR can more effectively shape equitable healthcare policies in Austria.

## 1 Introduction

In Austria, the healthcare system operates under a Bismarckian-type social health insurance model, which provides mandatory coverage for all residents and is characterized by a pluralistic structure of providers, payers, and regulators (1). Over the past two decades**,** several policy reforms have aimed to strengthen primary care and address inefficiencies arising from the historical emphasis on inpatient services (2). A key development is the introduction of primary healthcare units (PHCUs) through the Primary Care Act, which mandates the creation of localized “care strategies” and promotes interprofessional collaboration (3). These PHCUs are designed to offer comprehensive services—from preventative care to chronic disease management—to reduce fragmentation and increase the efficiency of the Austrian healthcare system.

Despite these reforms, the Austrian system continues to exhibit high rates of specialist consultations and hospital admissions in comparison to other developed countries, partly due to the absence of a gatekeeping mechanism (4, 5). This highlights a persistent need for more integrated, patient-centered approaches that emphasize disease management programs (6), mental health care (7), and community-based solutions, including home care support for older adults and individuals with dementia (8). Research also points to the growing role of digital health and telemedicine, especially as reflected by the rapid adoption of remote consultations during the COVID-19 pandemic (9). These evolving challenges underscore the importance of Health Services Research (HSR) in guiding evidence-based strategies, optimizing resource allocation, and ensuring equitable access to care.

Although numerous studies have examined particular facets of healthcare delivery in Austria—ranging from efficiency analyses of disease management programs (6, 10) to the experiences of mental health service users (11)**—**there remains a notable gap in comprehensive, bibliometric overviews of this research landscape. Such analyses can reveal publication trends, thematic priorities, and collaboration patterns, offering valuable insights for policymakers and healthcare stakeholders seeking to improve the quality, accessibility, and sustainability of healthcare services. Moreover, standardizing key terminologies (12) and conducting economic evaluations of primary care reforms (13) are increasingly recognized as vital steps toward a more cohesive and effective system.

Against this backdrop, the present study aims to map the evolution of HSR in Austria between 2000 and 2024, focusing on publication output, thematic foci, collaborative networks, and overall impact. By employing a bibliometric approach and drawing on integrated care principles (14) and health systems strengthening frameworks (15), we seek to (1) identify current research gaps, (2) highlight emerging priorities—such as digital health and PHCUs—and (3) propose future directions to foster an integrated and patient-centered healthcare system.

This study provides both theoretical and practical contributions to the field of health services research in Austria. From a theoretical perspective, it offers a detailed analysis of how research themes, collaboration networks, and publication trends have evolved over time, addressing gaps in the current literature. Practically, the findings highlight critical research gaps, such as the integration of digital health technologies and the need to improve healthcare access for underserved populations. These findings aim to guide research priorities and support policymakers in creating effective, evidence-based healthcare strategies. Building on these contributions, this study aspires to guide researchers, practitioners, and policymakers in harnessing the full potential of HSR to support ongoing reforms and promote equitable and sustainable health outcomes across Austria.

## 2 Methods

### 2.1 Study design

This bibliometric study aimed to map and analyze the evolution of health services research in Austria between 2000 and 2024. The research design in the search strategy followed systematic review principles (adapted from PRISMA guidelines) to provide a structured approach for article identification, screening, and data extraction. By focusing on publications with at least one author affiliated with an Austrian institution, the analysis sought to capture both the breadth and depth of locally relevant contributions to health services research.

### 2.2 Data sources and search strategy

A comprehensive search was performed on June 15, 2024, via the PubMed Advanced Search interface, which was chosen for its extensive coverage of biomedical and health-related literature. The search encompassed studies published from January 1, 2000, to July 31, 2024, ensuring that both historical and recent developments were represented. No language restrictions were applied, allowing the inclusion of articles in both English and German.

To balance specificity and sensitivity, the following Boolean formula was employed: “((Austria*[Affiliation]) AND ((“2000/01/01"[Date - Publication]: “3000"[Date - Publication]))) AND (health service*[Title/Abstract])”. This query yielded 422 results. Titles and abstracts were initially screened to confirm their relevance to health services research in the Austrian context. Publications that explicitly investigated topics such as healthcare delivery, organization, financing, or policy were considered potentially eligible and retained for further review.

### 2.3 Inclusion and exclusion criteria

**Figure 1:**
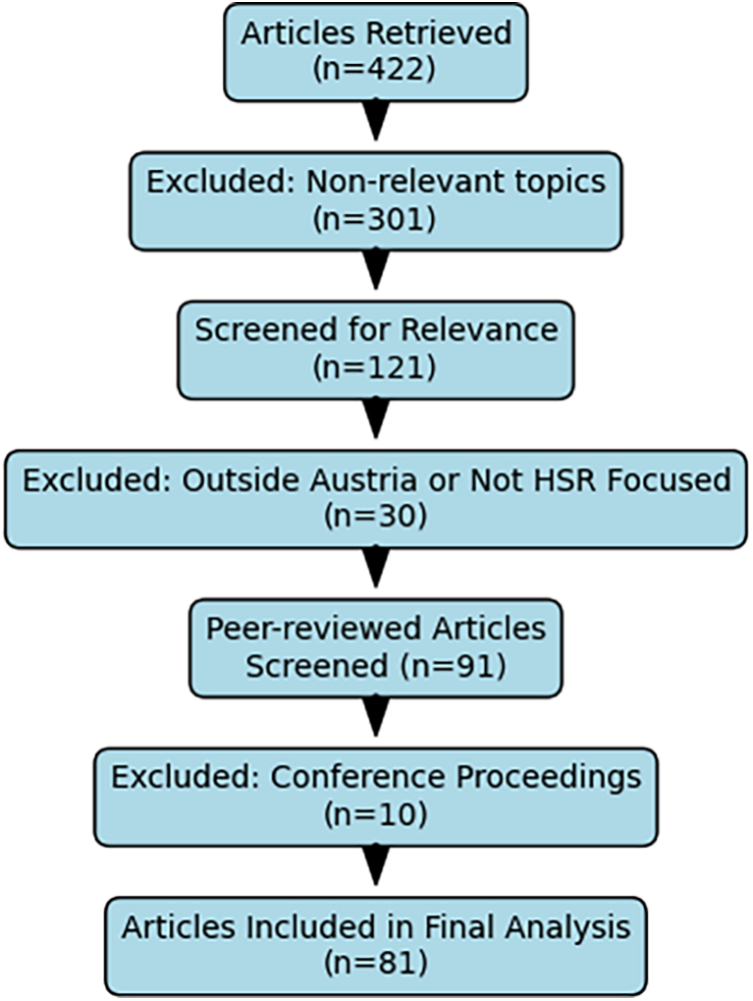
(PRISMA flow diagram) illustrates the progression from the initial retrieval of 422 articles to the final sample of 81 included in the analysis.

Eligible articles were those focusing on health services research in Austria, published within the specified timeframe, and having at least one author with a primary Austrian institutional affiliation. Publications in which fewer than 50% of the authors were affiliated with Austrian institutions were excluded, as were conference proceedings, to ensure the inclusion of peer-reviewed original articles. After title and abstract screening, full-text evaluations were conducted to confirm alignment with the study's focus on Austrian health services. Following the final eligibility check, 81 articles remained (Figure 1).

### 2.4 Data extraction and quality assurance

All the references were imported into Zotero (version 6.0.36) for systematic organization and deduplication. Two independent reviewers assessed the titles and abstracts; any disagreement was resolved through discussion or by consulting a third reviewer. Bibliographic details (title, authors, journal, publication year, DOI), Austrian institutional affiliations, and key thematic elements (e.g., chronic disease management, primary care models, mental health services) were extracted for each article.

The data were carefully cross-referenced with the original PubMed entries to resolve any discrepancies in publication years or author affiliations. To harmonize variations in author names and institutional names (e.g., “Med. Univ. Vienna” vs. “Medical University of Vienna”), we employed a hybrid approach that combined manual checks with semiautomated fuzzy string matching, implemented in Python (version 3.11.9). Specifically, the algorithm uses the Levenshtein distance via libraries such as pandas and fuzz (formerly fuzzywuzzy). Records exceeding a predefined similarity threshold (e.g., 0.85 on a scale of 0–1) were standardized to a uniform form (e.g., “Medical University of Vienna”). In cases where multiple high-scoring matches were identified or ambiguous terminology was encountered (e.g., “primary healthcare” vs. “primary health care”), manual review guaranteed consistent labeling.

These normalization procedures were critical for accurately capturing collaboration patterns and thematic clusters. These steps ensured a standardized dataset, forming the foundation for detailed analyses of collaboration networks and frequently occurring research themes—referred to here as thematic hotspots (e.g., chronic disease management).

### 2.5 Analysis

Descriptive statistical analyses, including trends in annual publication outputs and journal distribution, were conducted in Microsoft Excel (version 2019). Network analyses for coauthorship, institutional collaborations, and keyword cooccurrences were performed via VOSviewer (version 1.6.20) and Python. The visualizations generated in VOSviewer aided in identifying research clusters, thematic hotspots, and collaboration hubs, providing a detailed picture of Austria's health services research landscape.

### 2.6 Ethical considerations

This study relied solely on publicly accessible bibliographic data and did not involve human subjects or patient-level data; no ethical approval was needed. Nevertheless, certain limitations must be acknowledged. Relying on PubMed alone can exclude relevant articles indexed exclusively in other databases, such as Embase or Scopus, and excluding conference proceedings may omit emerging research not yet published in full. Additionally, the time frame cutoff of July 31, 2024, may have overlooked the latest publications. These limitations were deemed acceptable, given the study's objective to capture a broad, peer-reviewed snapshot of Austrian health services research over nearly a quarter century.

## 3 Results

### 3.1 Publication trends

A total of 81 articles met the inclusion criteria for this bibliometric exploration of health services research (HSR) in Austria between 2000 and 2024. The annual distribution of these articles showed an overall upward trend, with a pronounced increase in 2019 (9 articles) and 2020 (12 articles). This surge likely reflects intensified research efforts and global attention to healthcare systems during the onset of the COVID-19 pandemic, mirroring broader themes identified in related European contexts (4, 9). Nearly all the publications (96.3%) were original research articles, indicating a strong empirical orientation. The remainder comprised study protocols or observational reports (3.7%), indicating ongoing innovations in methodological development and real-world evidence generation.

**Figure 2:**
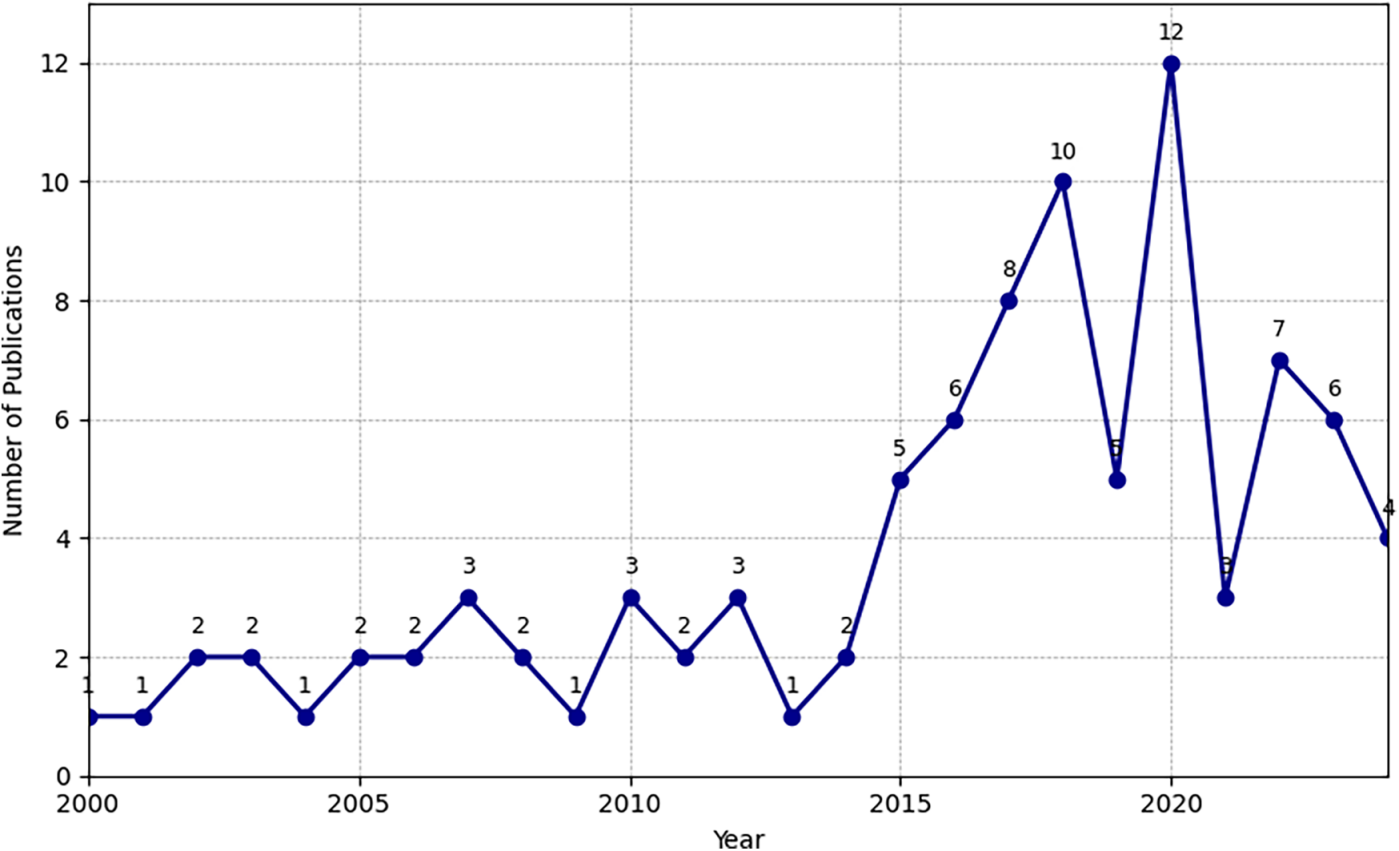
Number of publications on health services research in Austria (2000–2024).

Figure 2 presents the temporal distribution of publications between 2000 and 2024. Consistent with global shifts in healthcare priorities, studies have intensified around topics such as primary care models, mental health, and disease management programs (6, 16). The marked upticks in 2019 and 2020 were closely tied to the COVID-19 crisis, which catalyzed new lines of inquiry into service delivery, public health responses, and remote care strategies.

### 3.2 Methodological Spectrum

The study designs demonstrated considerable heterogeneity. Quantitative approaches were most common (55%), followed by qualitative (25%) and mixed-methods (20%). Approximately 60% of the articles reported collecting primary data, whereas 30% relied on secondary datasets (e.g., national registries such as the Austrian National CathLab Registry (17), administrative claims, or insurance databases). The remaining 10% offered no specification of data sources, highlighting occasional gaps in reporting transparency.

### 3.3 Thematic analysis

A longitudinal perspective on research themes revealed stable interest in patient care, public health, and integrated care frameworks, echoing Austria's broader system-level reforms (3). The prominence of mental health and psychiatry expanded notably after 2020, reflecting global shifts toward psychosocial well-being and mental health service integration (7, 11). Additionally, some studies have addressed the rollout of primary healthcare units (PHCUs) under the Austrian Primary Care Act, evaluating localized strategies to strengthen primary care provision (6).

**Figure 3:**
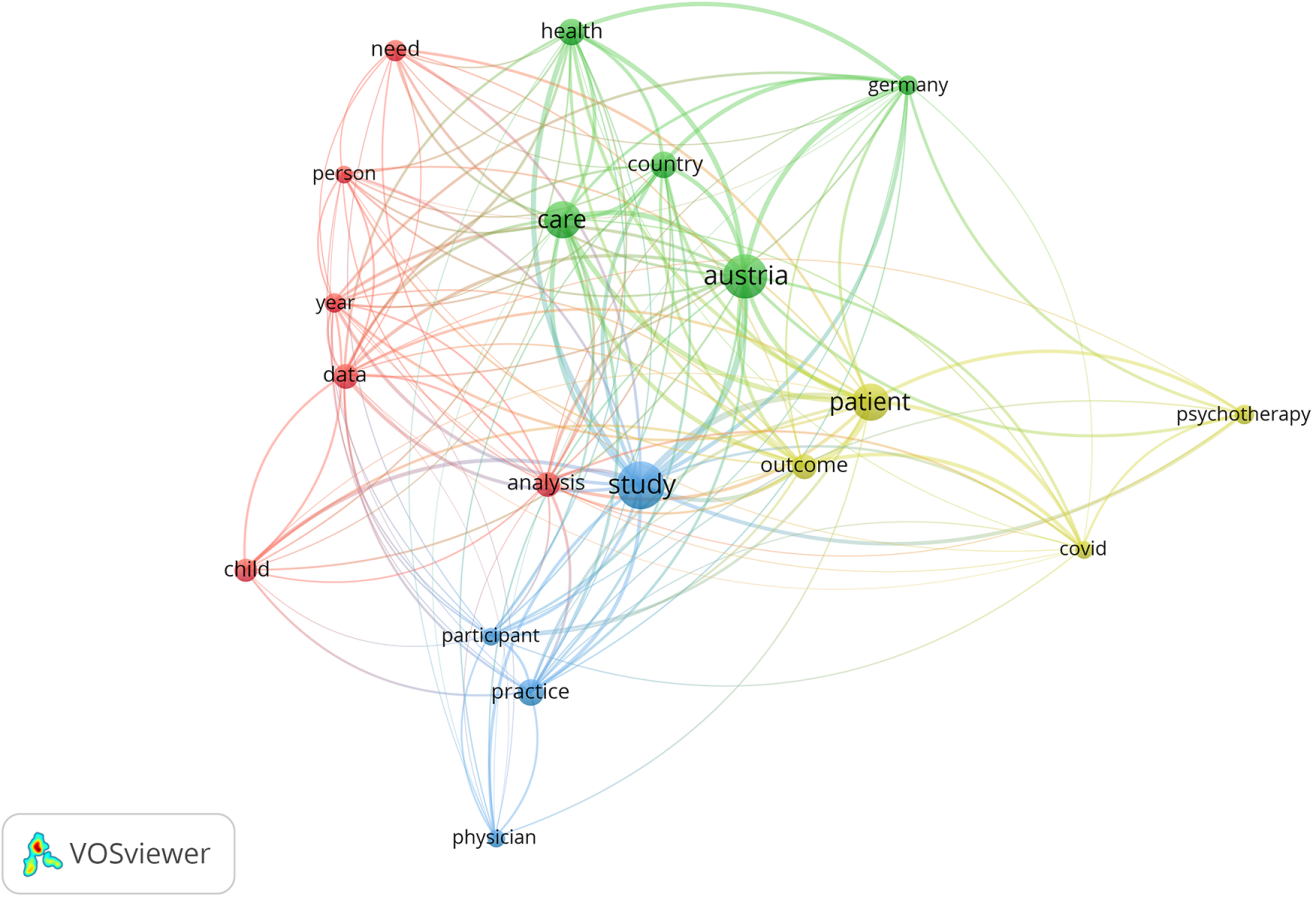
Keyword co-occurrence network for HSR in Austria.

Keyword co-occurrence analysis identified 19 frequent terms (≥10 occurrences). The most cited “study” (90), “Austria” (78), “care” (56), “patient” (54), and “health” (27) collectively underscored an enduring focus on healthcare delivery and patient-centered approaches. “Analysis” (25) highlights the data-driven nature of most studies, whereas “COVID” (13) underscores the pandemic's influence on Austrian research agendas. Figure 3 depicts these interrelated terms in a network layout, illustrating how mental health, patient care, and public health cluster alongside emerging interests such as telemedicine and digital health (9).

### 3.4 Collaboration and contribution networks

Coauthorship mapping demonstrated a diffuse yet collaborative research landscape, with no single dyad dominating. Authors such as Christoph Pieh, Elke Humer, and Kathryn Hoffmann appeared frequently and formed influential hubs in shaping Austrian HSR outputs. The analysis of institutional affiliations pinpointed the Medical University of Vienna as the most prolific contributor (8 publications), followed by the University for Continuing Education Krems (6 publications).

**Figure 4:**
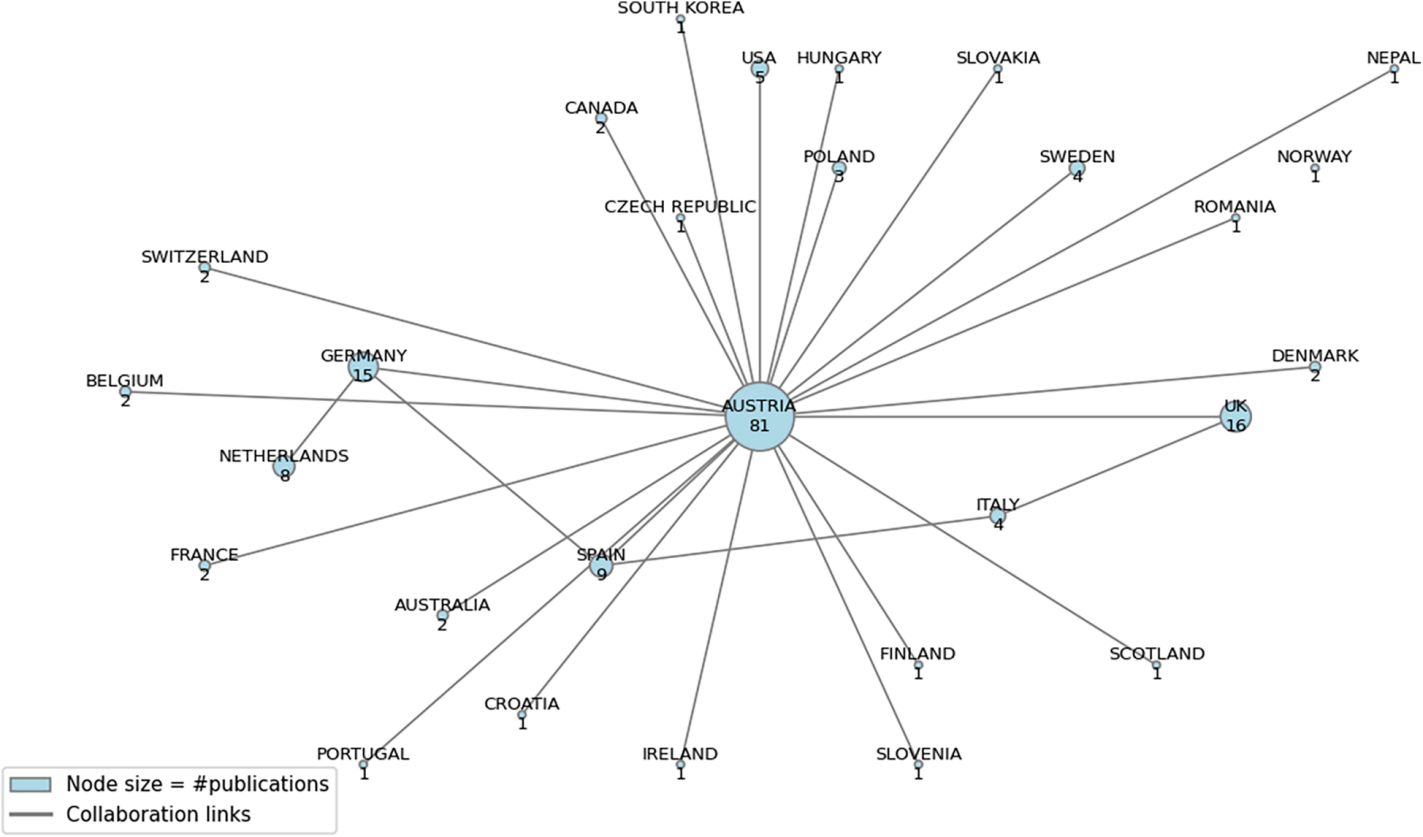
International collaboration networks in healthcare center research in Austria.

International partnerships were similarly robust, with the University of Liverpool and the University of Glasgow each coauthoring four articles. The top five authors—Hoffmann, Dorner, Wancata, Humer, and Pieh—produced four publications each, reflecting a tight-knit core of expertise. Figure 4 visually displays the contribution network, linking domestic institutions with European collaborators, especially from the United Kingdom, Germany, Spain, and Italy.

### 3.5 Geographical distribution and primary affiliation analysis

An affiliation-based assessment revealed that 43.32% of the articles originated from Austrian institutions, confirming the local relevance of the dataset (Table 1). Germany (7.49%) and the United Kingdom (5.88%) emerged as significant contributors. Betweenness centrality (BC) computations reinforced Austria's role as a key connector (BC = 0.381672), which is consistent with literature on Austria's active engagement in European consortia (e.g., EU-VIORMED, QUALICOPC).

**Table 1:** Top 10 countries contributing to research on healthcare centers in Austria based on author affiliations and betweenness centrality (BC).

Rank	Country	No. of publications	BC	Percentage (%)
1	Austria	81	0.381672	43.32
2	UK	16	0.077084	8.53
3	Germany	14	0.021528	7.49
4	Italy	7	0.049409	3.74
5	Netherlands	6	0.011994	3.21
6	Greece	5	0.020804	2.67
7	Norway	5	0.021871	2.67
8	Spain	5	0.020120	2.67
9	USA	5	0	2.67

### 3.6 Citation and impact analysis

Citation data from CrossRef, Web of Science, and Google Scholar highlighted a diverse range of impact among the 81 articles. The top five cited papers collectively addressed Hepatitis C drug pricing**,** mental health in adolescence, and online psychotherapy, with 96–509 citations each. Total citation counts peaked in 2016 (817), dominated by a high-impact examination of the costs and affordability of hepatitis C medications (Iyengar et al.), whereas a secondary increase occurred in 2020 (278), presumably linked to COVID-19-focused research. Table 2 shows the yearly distribution of aggregated citations from 2002 to 2024.

**Table 2:** Annual citation distribution in healthcare center research in Austria (2002–2024).

Publication year	Total citations
2002	29
2005	4
2006	25
2009	50
2010	47
2011	60
2012	151
2013	14
2014	51
2015	280
2016	817
2017	635
2018	252
2019	221
2020	278
2021	40
2022	88
2023	92
2024	2

### 3.7 Journal analysis

Most of the articles (78 of 81) were published in English, confirming an international dissemination strategy. BMJ Open was the top outlet (8 publications), followed by Wiener klinische Wochenschrift (7). Other influential venues included the European Journal of Public Health, Frontiers in Medicine, and BMC Health Services Research, each featuring two articles. The mean impact factor values were approximately 2.87 (SD = 1.85), whereas the average CiteScore was 3.40 (SD = 2.60), reflecting moderate- to high-impact publishing channels. Some journals, such as Frontiers in Medicine, had higher impact metrics (IF ∼5.091), indicating variable readership scopes.

**Figure 5:**
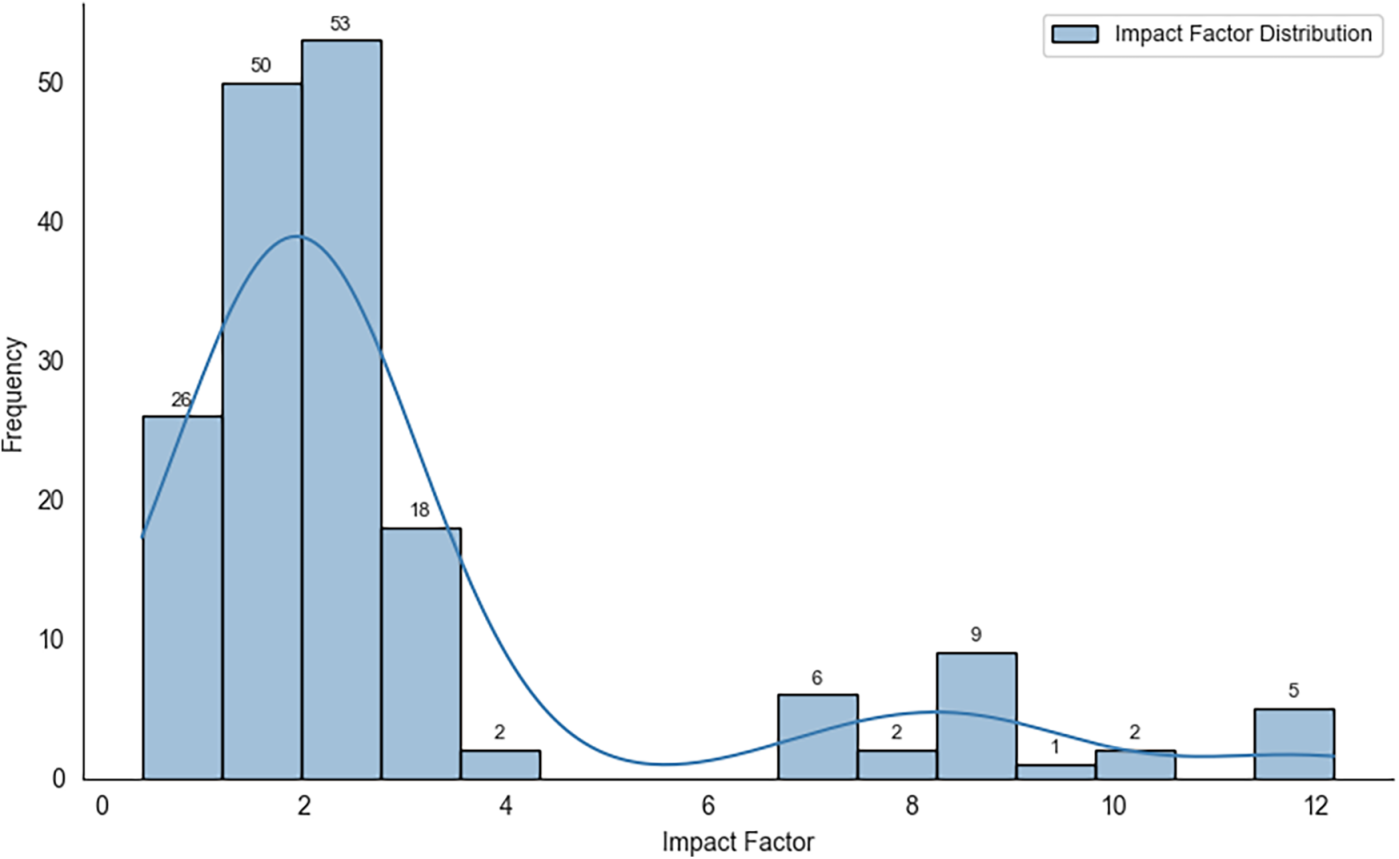
Distribution of impact factors for journals (years of publication).

Figure 5 shows the distribution of impact factors for the journals at the time of publication, revealing a right-skewed pattern with most outlets clustering around the 1–4 range. While many articles presented moderate citation rates, metric analyses have suggested stronger public or media engagement for certain publications.

Table 3 provides a comparative overview of journal metrics, including Impact Factor, CiteScore, SNIP, and SJR, illustrating how research impact varies across different publication venues.

**Table 3:** Comparative metrics for Key journals.

Journal	Impact factor	CiteScore	SNIP	SJR
European Journal of Preventive Cardiology	3.903	3.957	1.430	1.582
BMC Health Services Research	2.060	3.500	1.221	0.926
Neuropsychiatrie (Neuropsychiatr)	0.440	N/A	0.650	0.695
Wiener klinische Wochenschrift	0.829	1.900	0.454	0.204
Frontiers in Medicine	5.091	3.400	1.073	0.919

**Figure 6:**
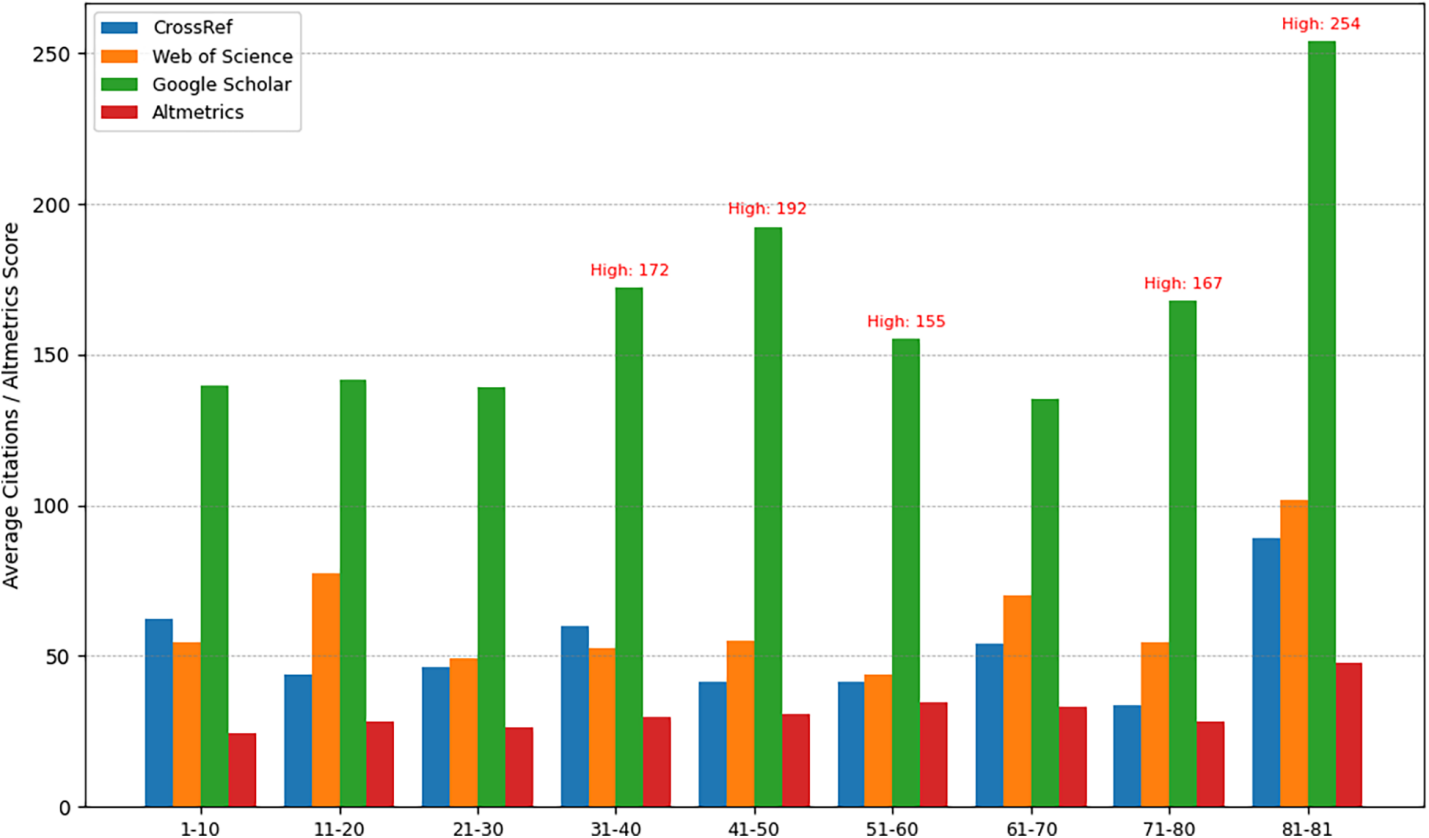
Metrics and altmetrics across articles.

Despite moderate citation rates in some instances, metric analyses (Figure 6) indicated higher public or media engagement for certain articles, highlighting the multidimensional nature of research impact.

### 3.8 Funding sources and conflicts of interest analysis

Disclosures regarding funding sources were heterogeneous. Among the 66 articles that mentioned financial support, 75.3% cited broad “other specific” funding—potentially from philanthropic, institutional, or nontraditional sources—while 18.5% did not mention any funding at all. Only five articles explicitly acknowledged commercial or not-for-profit backing (3.7%) or governmental grants (2.5%).

Conflict-of-interest statements mirrored this variability. The majority (85.2%) either omitted or vaguely addressed potential conflicts, with 3.7% explicitly declaring no conflicts and 8.6% acknowledging ties to industry or commissioning bodies (10, 18). Table 4 summarizes these patterns, suggesting that continued efforts to improve transparency may be warranted. Despite potential biases, such disclosures do not automatically invalidate findings but do underscore the importance of caution in interpreting outcomes tied to industry relationships.

**Table 4:** Distribution of funding sources in Austrian health services research.

Funding source type	Number of mentions
No explicit mention of specific funding sources	15
Commercial or not-for-profit sectors	3
Governmental funding	2
Other specific funding sources	61

Collectively, these findings depict an evolving and collaborative Austrian health services research domain shaped by national reforms (e.g., PHCUs) and international partnerships. Marked interest in mental health, patient-centered approaches, and the implications of the COVID-19 crisis align with global research priorities (19). Enhanced attention to disease management program evaluations (6) and economic analyses (13) signals a maturing field capable of informing evidence-based policy. Notwithstanding sporadic reporting gaps in funding and conflicts of interest, the surge in impactful publications and diverse thematic scopes highlights Austria's critical role in advancing health services research both regionally and internationally.

## 4 Discussion

This bibliometric analysis offers a comprehensive overview of health services research (HSR) in Austria from 2000 through 2024, underscoring the dynamic and evolving scholarly landscape. The notable increase in publications, especially from 2019 to 2020, parallels the heightened visibility of healthcare services globally amid the COVID-19 pandemic (4, 9). In the context of Austria, this period catalyzed empirical inquiries into disease management, remote care delivery, and pandemic preparedness, as exemplified by the predominance of original research articles (96.3%) focused on data-driven insights (10).

A balanced methodological palette emerged, comprising quantitative (55%), qualitative (25%), and mixed-methods (20%) studies. While quantitative designs elucidate large-scale patterns—e.g., healthcare utilization or outcomes—qualitative and mixed-methods research captures more nuanced patient perspectives (8, 11). The relatively higher prevalence of qualitative work in Austria indicates an emphasis on the human-centric dimensions of healthcare, aligning with legislative reforms aimed at strengthening patient-centered and integrated care (3).

The economic dimension surfaced frequently, although often as a secondary focus of patient care and public health. Evaluations of cost effectiveness, resource allocation, and long-term sustainability are nonetheless critical for shaping national policy (6, 13). Systematic economic analyses—particularly those related to Austrian primary healthcare units (PHCUs), disease management programs, or specialized outpatient care—could more fully inform both clinical practice and financial planning in Austria's Bismarckian insurance framework (1).

In addition to economic considerations, Austria's research landscape has increasingly prioritized mental health, reflecting broader global trends. This shift parallels imperatives to address psychosocial well-being and mental health burdens, exacerbated by pandemic-related disruptions (7). Several studies [e.g., (11)] highlight psychiatric rehospitalization experiences, illustrating Austria's growing recognition of mental health as integral to overall healthcare quality. In tandem, “COVID” emerged as a recurrent keyword, reflecting investigations into integrated health strategies (20), telehealth adoption (9), and psychosocial impacts on populations such as adolescents (21). These inquiries illuminate how public health crises reshape research priorities and health policy directives.

Beyond the thematic focus on mental health, Austria's active collaborations within the European research ecosystem have further shaped its methodological and policy directions. Institutions such as the Medical University of Vienna and partners in Germany and the UK have fostered cross-border healthcare innovations and methodological diversity (4). Citation metrics revealed heterogeneous impacts, with studies on drug pricing achieving high academic traction (22) and articles on public health topics gaining substantial engagement via Altmetrics. These complementary indicators underscore the importance of nuanced appraisals for both academic and societal influence.

Despite robust empirical outputs, inconsistent reporting of funding sources and conflicts of interest complicates the appraisal of potential biases. Enhanced transparency, particularly regarding industry sponsorship or governmental funding, bolsters credibility, public trust, and policy uptake (10, 18).

This study broadens the global HSR conversation by demonstrating a scalable hybrid methodology—integrating manual validation with automated methods—that can be adapted to diverse healthcare systems. For instance, Austria's approach to integrating digital health solutions in primary care could inform similar initiatives in countries transitioning toward patient-centered models. By leveraging these findings, nations can enhance their research landscapes and foster cross-border collaborations to address shared healthcare challenges. The hybrid methodology provides a replicable framework for analyzing complex datasets, enabling comparative research across healthcare systems globally.

### 4.1 Future directions

Several key gaps warrant further exploration. First, underserved populations—including rural residents, migrants, and older adults—remain relatively underrepresented, even though sociodemographic factors critically shape healthcare access and outcomes (19). Second, digital health and telemedicine are more systematically evaluated, despite the pandemic-driven acceleration of remote services (9). Evidence of clinical outcomes, patient satisfaction, and cost-effectiveness is vital for embedding telehealth into the routine continuum of care, particularly in remote or underserved regions. Additionally, the economic and structural sustainability of newly introduced PHCUs or other coordinated outpatient models demands rigorous assessment, ensuring that improvements in accessibility and continuity of care do not compromise financial viability (3).

More broadly, addressing issues such as gatekeeping (5), wherein unrestricted specialist access can inflate costs, could complement Austria's reforms targeting primary care strength. Comparative studies with systems such as Germany or the UK (4) might elucidate policy levers for equitable and efficient healthcare delivery.

### 4.2 Limitations of the study

This bibliometric analysis relies exclusively on PubMed, potentially overlooking relevant contributions indexed in Embase, Scopus, or nonindexed local journals. Additionally, focusing on traditional metrics (Impact Factor, CiteScore) may underrepresent broader societal or policy influences, suggesting that future reviews incorporate multiple measures, including Altmetrics, the h-index, and the Eigenfactor. Finally, inconsistent reporting of funding and conflicts of interest introduces uncertainty about potential biases. Although these constraints do not diminish the core insights presented here, they underscore the need for multisource searching and transparent disclosures in subsequent bibliometric research.

## 5 Conclusion

This bibliometric study maps the trajectory of Austrian health services research between 2000 and 2024, highlighting the importance of mental health, patient-centered initiatives, and digital healthcare solutions alongside established emphases on disease management and public health. Austria's centrally positioned role in collaborative European networks supports methodological innovation and the generation of policy-relevant evidence. However, inconsistent transparency regarding funding and conflict-of-interest disclosures highlights opportunities for improving research integrity. Moving forward, addressing identified gaps—particularly around underserved populations, the effectiveness of telemedicine, and the economic sustainability of primary care reforms—will be crucial to leveraging HSR for equitable, evidence-based healthcare development across Austria.

## Data Availability

The original contributions presented in the study are included in the article/Supplementary Material, further inquiries can be directed to the corresponding author.
