# Supplementary material for: The evolution of health services research in Austria: a bibliometric exploration of trends, themes, and collaborations
Source: Front Health Serv. 2025 Mar 13;5:1501035. doi: 10.3389/frhs.2025.1501035 (PMC11966452; doi:10.3389/frhs.2025.1501035)
Supplement: Supplementary file 2 [file Datasheet1.pdf]

Author	Title	Year	Title	DOI	Url
Adlbrecht, Christopher; Neuhold, Stephanie; Hülsmann, Martin; Strunk, Guido; Ehmsen, Udo; Scholten, Christine; Maurer, Gerald; Pacher, Richard	NT-proBNP as a means of triage for the risk of hospitalisation in primary care	2012	European Journal of Preventive Cardiology	10.1177/1741826710391545	https://academic.oup.com/eurjpc/article/19/1/55-61/5928265
Ådnanes, M.; Melby, L.; Cresswell-Smith, J.; Westerlund, H.; Rabbi, L.; Dernovšek, M. Z.; Šprah, L.; Sfetcu, R.; Straßmayr, C.; Donisi, V.	Mental health service users' experiences of psychiatric re-hospitalisation - an explorative focus group study in six European countries	2018	BMC Health Services Research	10.1186/s12913-018-3317-1	https://bmchealthservres.biomedcentral.com/articles/10.1186/s12913-018-3317-1
Alexandrowicz, Rainer W.; Bacher, Johann; Wancata, Johannes	Sampling and weighting of the Austrian Psychiatric Prevalence Survey (APPS)	2019	neuropsychiatrie	10.1007/s40211-019-0305-6	http://link.springer.com/10.1007/s40211-019-0305-6
Mühlberger, Volker; Kobel, Conrad; Kaltenbach, Lalit; Pachinger, Otmar	Austrian National CathLab Registry (ANCALAR): cardiac catheterization, coronary angiography (CA), and percutaneous coronary intervention (PCI) in Austria during the year 2011 (Registry Data with Audit including 2012)	2013	Wiener klinische Wochenschrift	10.1007/s00508-013-0459-3	http://link.springer.com/10.1007/s00508-013-0459-3
Borrmann, Marcus; Lindner, Sonja; Hofer-Fischanger, Kathrin; Rehb, Robert; Pechstädt, Katrin;	Strategy for Deployment of Integrated Healthy Aging Regions Based	2020	Frontiers in Medicine	10.3389/fmed.2020.510475	https://www.frontiersin.org/article/10.33

Wiedenhofer, Roswitha; Schwarze, Gabriele; Adamer-König, Eva-Maria; Mischak, Robert; Pfeiffer, Karl P.; Harer, Johann; Weinzerl, Katharina; Hartmann, Christian; Rupp, Bernhard; Roller-Wirnsberger, Regina E.	Upon an Evidence-Based Regional Ecosystem—The Styria Model				89/fmed.2020.510475/full
Brettell, Elizabeth; Högler, Wolfgang; Woolley, Rebecca; Cummins, Carole; Mathers, Jonathan; Oppong, Raymond; Roy, Laura; Khan, Adam; Hunt, Charmaine; Dattani, Mehul; on behalf of the G. H. D. study group; Ong, Ken; Donaldson, Malcolm; Harris, Victoria; Maghnie, Mohamad; Gregory, John; Auguste, Peter; Binder, Gerhard; Gambol, Carrol; Dhamaraj, Poonam; Gevers, Evelien; Saraff, Vrinda; Clayton, Peter; Randell, Tabitha; Mushtaq, Talat; Cheetham, Timothy; Davies, Justin; Abid, Noina; Khairi, Ranna El; Kapelari, Klaus; Gottardi-Butturini, Elena; Reiterer-Fröhlich, Elke; Bonfig, Walter	The Growth Hormone Deficiency (GHD) Reversal Trial: effect on final height of discontinuation versus continuation of growth hormone treatment in pubertal children with isolated GHD—a non-inferiority Randomised Controlled Trial (RCT)	2023	Trials	10.1186/s13063-023-07562-z	https://trialsjournal.biomedcentral.com/articles/10.1186/s13063-023-07562-z
Buchholz, Maresa; Weber, Niklas; Borel, Stephanie; Sayah, Sabrina; Xie, Feng; Schulz, Jörg B; Reetz, Kathrin; Boesch, Sylvia; Klopstock, Thomas; Karin, Ivan; Schöls,	Patient-reported, health economic and psychosocial outcomes in patients with Friedreich ataxia (PROFA): protocol of an	2023	BMJ Open	10.1136/bmjopen-2023-075736	https://bmjopen.bmj.com/lookup/doi/10.1136/bmjopen-2023-

Ludger; Grobe-Einsler, Marcus; Klockgether, Thomas; Davies, Elin Haf; Schmeder, Madeleine; Nadke, Andreas; Michalowsky, Bernhard	observational study using momentary data assessments via mobile health app				075736
Carmichael, Christina; Schiffler, Tobias; Smith, Lee; Moudatsou, Maria; Tabaki, Ioanna; Doñate-Martínez, Ascensión; Alhambra-Borrás, Tamara; Kouvari, Matina; Karnaki, Pania; Gil-Salmeron, Alejandro; Grabovac, Igor	Barriers and facilitators to health care access for people experiencing homelessness in four European countries: an exploratory qualitative study	2023	International Journal for Equity in Health	10.1186/s12939-023-02011-4	https://equityhealthj.biomedcentral.com/articles/10.1186/s12939-023-02011-4
Clar, Clemens; Koutp, Amir; Leithner, Andreas; Leitner, Lukas; Puchwein, Paul; Vielgut, Ines; Sadoghi, Patrick	Occupational injuries in orthopedic and trauma surgeons in Austria	2024	Archives of Orthopaedic and Trauma Surgery	10.1007/s00402-024-05200-0	https://link.springer.com/10.1007/s00402-024-05200-0
De Brún, T.; O'Reilly - De Brún, M.; Van Weel-Baumgarten, E.; Burns, N.; Dowrick, C.; Lionis, C.; O'Donnell, C.; Mair, F. S.; Papadakaki, M.; Saridaki, A.; Spiegel, W.; Van Weel, C.; Van Den Muijsenbergh, M.; MacFarlane, A.	Using Participatory Learning & Action (PLA) research techniques for inter-stakeholder dialogue in primary healthcare: an analysis of stakeholders' experiences	2017	Research Involvement and Engagement	10.1186/s40900-017-0077-8	https://researchinvolvement.biomedcentral.com/articles/10.1186/s40900-017-0077-8
De Girolamo, Giovanni; Iozzino, Laura; Ferrari, Clarissa; Gosek, Pawel; Heitzman, Janusz; Salize, Hans Joachim; Wancata, Johannes; Picchioni, Marco; Macis, Ambra	A multinational case–control study comparing forensic and non-forensic patients with schizophrenia spectrum disorders: the EU-VIORMED project	2023	Psychological Medicine	10.1017/S0033291721003433	https://www.cambridge.org/core/product/identifier/S0033291721003433/type/journal_article

Plunger, Petra; Heimerl, Katharina; Tatzer, Verena C; Zepke, Georg; Finsterwald, Monika; Pichler, Barbara; Reitinger, Elisabeth	Developing dementia-friendly pharmacies in Austria: a health promotion approach	2020	Health Promotion International	10.1093/heapro/daz063	https://academic.oup.com/heapro/article/35/4/702/5530704
Spaemann, Christian	Die Rolle der psychiatrischen Fachabteilung am Allgemeinkrankenhaus in der gemeindepsychiatrischen Versorgung	2006	Wiener Medizinische Wochenschrift	10.1007/s10354-005-0252-7	https://link.springer.com/10.1007/s10354-005-0252-7
Diermayr, Gudrun; Schomberg, Maria; Greisberger, Andrea; Elsner, Bernhard; Gronwald, Marit; Salbach, Nancy M	Task-Oriented Circuit Training for Mobility in Outpatient Stroke Rehabilitation in Germany and Austria: A Contextual Transferability Analysis	2020	Physical Therapy	10.1093/ptj/pzaa053	https://academic.oup.com/ptj/article/100/8/1307/5816581
Fellinger, Matthäus; Knasmüller, Philipp; Kocsis-Bogar, Krisztina; Wippel, Andreas; Fragner, Laura; Mairhofer, Dunja; Hochgatterer, Paulus; Aigner, Martin	Adverse childhood experiences as risk factors for recurrent admissions in young psychiatric inpatients	2022	Frontiers in Psychiatry	10.3389/fpsyt.2022.988695	https://www.frontiersin.org/articles/10.3389/fpsyt.2022.988695/full
Fuchs, Martin; Kemmler, Georg; Steiner, Hans; Marksteiner, Josef; Haring, Christian; Miller, Carl; Hausmann, Armand; Sevecke, Kathrin	Child and adolescent psychiatry patients coming of age: a retrospective longitudinal study of inpatient treatment in Tyrol	2016	BMC Psychiatry	10.1186/s12888-016-0910-x	http://bmcpsychiatry.biomedcentral.com/articles/10.1186/s12888-016-0910-x
Georges, Daniela; Buber-Ennser, Isabella; Rengs, Bernhard; Kohlenberger, Judith;	Health determinants among refugees in Austria and	2021	PLOS ONE	10.1371/journal.pone250821	https://dx.plos.org/10.1371/journ

Doblhammer, Gabriele	Germany: A propensity-matched comparative study for Syrian, Afghan, and Iraqi refugees				al.pone250821
Gothe, Holger; Matteucci Gothe, Raffaella; Arvandi, Marjan; Hintringer, Katharina; Toell, Thomas; Oberaigner, Willi; Rajsic, Sasa; Kugler, Joachim; Kiechl, Stefan; Willeit, Johann; Siebert, Uwe	Linkage von klinischen Primärdaten und Krankenkassenabrechnungsdaten in der Evaluation der Schlaganfallversorgung – SeDaStro: Erfahrungen aus dem Tiroler StrokeCard-Programm	2020	Das Gesundheitswesen	10.1055/a-1101-8949	http://www.thieme-connect.de/DOI/DOI?10.1055/a-1101-8949
Haluza, Daniela; Hofer, Fanni	Exploring perceptions on medical app use in clinical communication among Austrian physicians: Results of a validation study	2020	Health Informatics Journal	10.1177/1460458219888420	http://journals.sagepub.com/doi/10.1177/1460458219888420
Niederkrotenthaler, Thomas; Mittendorfer-Rutz, Ellenor; Thurner, Stefan; Endel, Gottfried; Klimek, Peter	Healthcare utilization, psychiatric medication and risk of rehospitalization in suicide-attempting patients with common mental disorders	2020	Australian & New Zealand Journal of Psychiatry	10.1177/0004867419895112	http://journals.sagepub.com/doi/10.1177/0004867419895112
Heinze, Georg; Wallisch, Christine; Kainz, Alexander; Hronsky, Milan; Leffondré, Karen; Oberbauer, Rainer; Mayer, Gert	Chances and challenges of using routine data collections for renal health care research	2015	Nephrology Dialysis Transplantation	10.1093/ndt/gfv110	https://academic.oup.com/ndt/article-lookup/doi/10.1093/ndt/gfv110

Hoffmann, Kathryn; Wojczewski, Silvia; Aarendonk, Diederik; Maier, Manfred; Dorner, Thomas Ernst; De Maeseneer, Jan	No common understanding of profession terms utilized in health services research: An add-on qualitative study in the context of the QUALICOPC project in Austria	2017	Wiener klinische Wochenschrift	10.1007/s00508-016-1146-y	http://link.springer.com/10.1007/s00508-016-1146-y
Hoffmann, Kathryn; George, Aaron; Jirovsky, Elena; Dorner, Thomas E	Re-examining access points to the different levels of health care: a cross-sectional series in Austria	2019	European Journal of Public Health	10.1093/eurpub/ckz050	https://academic.oup.com/eurpub/article/29/6/1005/5425251
Hoffmann, Kathryn; Ristl, Robin; George, Aaron; Maier, Manfred; Pichlhöfer, Otto	The ecology of medical care: access points to the health care system in Austria and other developed countries	2019	Scandinavian Journal of Primary Health Care	10.1080/02813432.2019.1663593	https://www.tandfonline.com/doi/full/10.1080/02813432.2019.1663593
Hummer, Michael; Lehner, Thomas; Pruckner, Gerald	Low birth weight and health expenditures from birth to late adolescence	2014	The European Journal of Health Economics	10.1007/s10198-013-0468-1	http://link.springer.com/10.1007/s10198-013-0468-1
Zechmeister, Ingrid; Österle, August; Denk, Peter; Katschnig, Heinz	Incentives in Financing Mental Health Care in Austria	2002	J Ment Health Policy Econ	nan	nan
Vyssoki, B.; Willeit, M.; Blüml, V.; Höfer, P.; Erfurth, A.; Psota, G.; Lesch, O.M.; Kapusta, N.D.	Inpatient treatment of major depression in Austria between 1989 and 2009: Impact of downsizing of psychiatric hospitals on admissions,	2011	Journal of Affective Disorders	10.1016/j.jad.2011331	https://linkinghub.elsevier.com/retrieve/pii/S0165032711001303

	suicide rates and outpatient psychiatric services				
Berghold, Christian; Berghold, Andrea; Fülöp, Gerhard; Heuberger, Sigrid; Strauss, Reinhild; Zenz, Werner	Invasive meningococcal disease in Austria 2002: assessment of completeness of notification by comparison of two independent data sources	2006	Wiener klinische Wochenschrift	10.1007/s00508-005-0502-0	https://link.springer.com/10.1007/s00508-005-0502-0
Iyengar, Swathi; Tay-Teo, Kiu; Vogler, Sabine; Beyer, Peter; Wiktor, Stefan; De Joncheere, Kees; Hill, Suzanne	Prices, Costs, and Affordability of New Medicines for Hepatitis C in 30 Countries: An Economic Analysis	2016	PLOS Medicine	10.1371/journal.pmed.1002032	https://dx.plos.org/10.1371/journal.pmed.1002032
Jesser, Andrea; Schaffler, Yvonne; Gächter, Afsaneh; Dale, Rachel; Humer, Elke; Pieh, Christoph	School Students' Concerns and Support after One Year of COVID-19 in Austria: A Qualitative Study Using Content Analysis	2022	Healthcare	10.3390/healthcare10071334	https://www.mdpi.com/2227-9032/10/7/1334
Kapusta, Nestor D; Posch, Martin; Niederkrotenthaler, Thomas; Fischer-Kern, Melitta; Etzersdorfer, Elmar; Sonneck, Gernot	Availability of Mental Health Service Providers and Suicide Rates in Austria: A Nationwide Study	2010	nan	nan	nan
Kautzky-Willer, Alexandra; Kaleta, Michaela; Lindner, Simon D.; Leutner, Michael; Thurner, Stefan; Klimek, Peter	Sex Differences in Clinical Characteristics and Outcomes of Patients with SARS-CoV-2-Infection Admitted to Intensive Care Units in Austria	2022	Journal of Personalized Medicine	10.3390/jpm12040517	https://www.mdpi.com/2075-4426/12/4/517
Korecka, Nicole; Rabenstein, Rafael;	Psychotherapy by Telephone or	2020	International Journal of	10.3390/ijerph1721	https://www.md

Pieh, Christoph; Stippl, Peter; Barke, Antonia; Doering, Bettina; Gossmann, Katharina; Humer, Elke; Probst, Thomas	Internet in Austria and Germany Which CBT Psychotherapists Rate It more Comparable to Face-to-Face Psychotherapy in Personal Contact and Have more Positive Actual Experiences Compared to Previous Expectations?		Environmental Research and Public Health	7756	pi.com/1660-4601/17/21/7756
Kreidl, P.; De Kat, Catharina; Luckner-Hornischer, A.; Decristoforo, P.; Broksch, I.; Würzner, R.; Schmid, Daniela	Utilization and impact of European immunization week to increase measles, mumps, rubella vaccine uptake in Austria in 2016	2017	Vaccine	10.1016/j.vaccine.2017747	https://linkinghub.elsevier.com/retrieve/pii/S0264410X17309556
Kronbichler, Andreas; Effenberger, Maria; Shin, Jae Il; Koppelstätter, Christian; Denicolò, Sara; Rudnicki, Michael; Neuwirt, Hannes; Soler, Maria José; Stevens, Kate; Bruchfeld, Annette; Tilg, Herbert; Mayer, Gert; Perco, Paul	Is There Decreasing Public Interest in Renal Transplantation? A Google TrendsTM Analysis	2020	Journal of Clinical Medicine	10.3390/jcm9041048	https://www.mdpi.com/2077-0383/9/4/1048
Krutter, Simon; Schaffler-Schaden, Dagmar; Eßl-Maurer, Roland; Seymer, Alexander; Osterbrink, Juergen; Flamm, Maria	Home care nursing for persons with dementia from a family caregivers' point of view: Predictors of utilisation in a rural setting in Austria	2022	Health & Social Care in the Community	10.1111/hsc.13412	https://onlinelibrary.wiley.com/doi/10.1111/hsc.13412
Łaszewska, Agata; Schwab, Markus; Leutner, Eva;	Measuring broader wellbeing in	2019	Quality of Life Research	10.1007/s11136-019-	http://link.springer.com/

Oberrauter, Marold; Spiel, Georg; Simon, Judit	mental health services: validity of the German language OxCAP-MH capability instrument			02187-9	10.1007/s11136-019-02187-9
Łaszewska, Agata; Wancata, Johannes; Jahn, Rebecca; Simon, Judit	The excess economic burden of mental disorders: findings from a cross-sectional prevalence survey in Austria	2020	The European Journal of Health Economics	10.1007/s10198-020-01200-0	https://link.springer.com/10.1007/s10198-020-01200-0
Lionis, Christos; Papadakaki, Maria; Saridaki, Aristoula; Dowrick, Christopher; O'Donnell, Catherine A; Mair, Frances S; Van Den Muijsenbergh, Maria; Burns, Nicola; De Brún, Tomas; O'Reilly De Brún, Mary; Van Weel-Baumgarten, Evelyn; Spiegel, Wolfgang; MacFarlane, Anne	Engaging migrants and other stakeholders to improve communication in cross-cultural consultation in primary care: a theoretically informed participatory study	2016	BMJ Open	10.1136/bmjopen-2015-010822	https://bmjopen.bmj.com/lookup/doi/10.1136/bmjopen-2015-010822
Mahlknecht, Angelika; Engl, Adolf; Barbieri, Verena; Bachler, Herbert; Obwegeser, Alois; Piccoliori, Giuliano; Wiedermann, Christian J.	Attitudes towards career choice and general practice: a cross-sectional survey of medical students and residents in Tyrol, Austria	2024	BMC Medical Education	10.1186/s12909-024-05205-8	https://bmcmeduc.biomedcentral.com/articles/10.1186/s12909-024-05205-8
Mathis-Edenhofer, Stefan; Röthlin, Florian; Wachabauer, David; Haneef, Romana; Ventura, Ilana; Fülöp, Gerhard	Regional health care profiles – an improved method for generating case studies on the catchment areas of envisaged primary health care units in Austria: a report to the InfAct Joint	2022	Archives of Public Health	10.1186/s13690-022-00821-6	https://archpublichealth.biomedcentral.com/articles/10.1186/s13690-022-00821-6

	Action				
Mayer, Susanne; Österle, August	Socioeconomic determinants of prescribed and non-prescribed medicine consumption in Austria	2015	The European Journal of Public Health	10.1093/eurpub/cku179	https://academic.oup.com/eurpub/article-lookup/doi/10.1093/eurpub/cku179
Munsch, Nicolas; Gruarin, Stefanie; Nateqi, Jama; Lutz, Thomas; Binder, Michael; Aberle, Judith H.; Martin, Alistair; Knapp, Bernhard	Symptoms associated with a COVID-19 infection among a non-hospitalized cohort in Vienna	2022	Wiener klinische Wochenschrift	10.1007/s00508-022-02028-9	https://link.springer.com/10.1007/s00508-022-02028-9
Oberndorfer, R.; Alexandrowicz, R. W.; Unger, A.; Koch, M.; Markiewicz, I.; Gosek, P.; Heitzman, J.; Iozzino, L.; Ferrari, C.; Salize, H.-J.; Picchioni, M.; Fangerau, H.; Stompe, T.; Wancata, J.; De Girolamo, G.	Needs of forensic psychiatric patients with schizophrenia in five European countries	2023	Social Psychiatry and Psychiatric Epidemiology	10.1007/s00127-022-02336-5	https://link.springer.com/10.1007/s00127-022-02336-5
O'Donnell, Catherine A; Mair, Frances S; Dowrick, Christopher; Brún, Mary O'Reilly-de; Brún, Tomas De; Burns, Nicola; Lionis, Christos; Saridaki, Aristoula; Papadakaki, Maria; Muijsenbergh, Maria Van Den; Weel-Baumgarten, Evelyn Van; Gravenhorst, Katja; Cooper, Lucy; Princz, Christine; Teunissen, Erik; Mareeuw, Francine Van Den Driessen; Vlahadi, Maria; Spiegel, Wolfgang; MacFarlane, Anne	Supporting the use of theory in cross-country health services research: a participatory qualitative approach using Normalisation Process Theory as an example	2017	BMJ Open	10.1136/bmjopen-2016-014289	https://bmjopen.bmj.com/lookup/doi/10.1136/bmjopen-2016-014289
Zurl, Brigitte; Bayerl,	ÖGRO survey on	2018	Strahlenther	10.1007/s	http://li

Anja; De Vries, Alexander; Geinitz, Hans; Hawliczek, Robert; Knocke-Abulesz, Tomas-Henrik; Lukas, Peter; Pötter, Richard; Raunik, Wolfgang; Scholz, Brigitte; Schratter-Sehn, Annemarie; Sedlmayer, Felix; Seewald, Dietmar; Selzer, Edgar; Kapp, Karin S.	radiotherapy capacity in Austria: Status quo and estimation of future demands		apie und Onkologie	00066-017-1240-8	nk.springer.com/10.1007/s00066-017-1240-8
Ostermann, Herwig; Hoess, Victoria; Mueller, Michael	Efficiency of the Austrian disease management program for diabetes mellitus type 2: a historic cohort study based on health insurance provider's routine data	2012	BMC Public Health	10.1186/1471-2458-12-490	http://bmcpublichealth.biomedcentral.com/articles/10.1186/1471-2458-12-490
Piribauer, Franz; Thaler, Kylie; Harris, Mark F	Covert checks by standardised patients of general practitioners' delivery of new periodic health examinations: clustered cross-sectional study from a consumer organisation	2012	BMJ Open	10.1136/bmjopen-2011-000744	https://bmjopen.bmj.com/lookup/doi/10.1136/bmjopen-2011-000744
Poeppl, Wolfgang; Herkner, Harald; Burgmann, Heinz; Pustelnik, Tom; Mooseder, Gerhard; Popow-Kraupp, Theresia; Redlberger-Fritz, Monika	Performance of the QuickVue Influenza A+B Rapid Test for Pandemic H1N1 (2009) Virus Infection in Adults	2011	PLoS ONE	10.1371/journal.pone.0028089	https://journals.plos.org/plosone/article?id=10.1371/journal.pone.0028089
Poggenburg, Stephanie; Reinisch, Manuel; Höfler, Reinhild; Stigler, Florian; Avian,	General practitioners in Styria – who is willing to take	2017	Wiener klinische Wochenschrift	10.1007/s00508-017-1244-5	https://link.springer.com/article/1

Alexander; Siebenhofer, Andrea	part in research projects and why?: A survey by the Institute of General Practice and Health Services Research				0.1007/s00508-017-1244-5
Puchner, Rudolf; Vavrovsky, Anna; Pieringer, Herwig; Hochreiter, Ronald; Machold, Klaus P.	The Supply of Rheumatology Specialist Care in Real Life. Results of a Nationwide Survey and Analysis of Supply and Needs	2020	Frontiers in Medicine	10.3389/fmed.20200016	https://www.frontiersin.org/journals/medicine/articles/10.3389/fmed.2020.00016/full
Rabady, Susanne; Hoffmann, Kathryn; Brose, Markus; Lammel, Oliver; Poggenburg, Stefanie; Redlberger-Fritz, Monika; Stiasny, Karin; Wendler, Maria; Weseslindtner, Lukas; Zehetmayer, Sonja; Kamenski, Gustav	Symptoms and risk factors for hospitalization of COVID-19 presented in primary care: An exploratory retrospective study	2022	Wiener klinische Wochenschrift	10.1007/s00508-021-01992-y	https://link.springer.com/10.1007/s00508-021-01992-y
Zechmeister-Koss, Ingrid; Tüchler, Heinz; Goodyear, Melinda; Lund, Ingunn Olea; Paul, Jean Lillian	Reaching families where a parent has a mental disorder: Using big data to plan early interventions	2020	neuropsychiatrie	10.1007/s40211-019-00323-y	http://link.springer.com/10.1007/s40211-019-00323-y
Reiter, Julia; Weibelzahl, Stephan; Duden, Gesa S	Would've, could've, should've: a cross-sectional investigation of whether and how healthcare staff's working conditions and mental health symptoms have changed throughout 3	2024	BMJ Open	10.1136/bmjopen-2023-076712	https://bmjopen.bmj.com/lookup/doi/10.1136/bmjopen-2023-076712

	pandemic years				
Rinner, Christoph; Sauter, Simone Katja; Endel, Gottfried; Heinze, Georg; Thurner, Stefan; Klimek, Peter; Duftschmid, Georg	Improving the informational continuity of care in diabetes mellitus treatment with a nationwide Shared EHR system: Estimates from Austrian claims data	2016	International Journal of Medical Informatics	10.1016/j.ijmedinf.2016501	https://linkinghub.elsevier.com/retrieve/pii/S1386505616300739
Rutten-van Mölken, Maureen; Karimi, Milad; Leijten, Fenna; Hoedemakers, Maaike; Looman, Willemijn; Islam, Kamrul; Askildsen, Jan E; Kraus, Markus; Ercevic, Darija; Struckmann, Verena; Gyorgy Pitter, János; Cano, Isaac; Stokes, Jonathan; Jonker, Marcel	Comparing patients' and other stakeholders' preferences for outcomes of integrated care for multimorbidity: a discrete choice experiment in eight European countries	2020	BMJ Open	10.1136/bmjopen-2020-037547	https://bmjopen.bmj.com/lookup/doi/10.1136/bmjopen-2020-037547
Salvador-Carulla, Luis; Amaddeo, Francesco; Gutiérrez-Colosía, Mencía R.; Salazzari, Damiano; Gonzalez-Caballero, Juan Luis; Montagni, Ilaria; Tedeschi, Federico; Cetrano, Gaia; Chevreul, Karine; Kalseth, Jorid; Hagmair, Gisela; Straßmayr, Christa; Park, A-La; Sfetcu, Raluca; Wahlbeck, Kristian; Garcia-Alonso, Carlos	Developing a tool for mapping adult mental health care provision in Europe: the REMAST research protocol and its contribution to better integrated care	2015	International Journal of Integrated Care	10.5334/ijic.2417	http://www.ijic.org/article/10.5334/ijic.2417/
Santillan-Ramos, Paola; Humer, Elke; Schaffler, Yvonne; Pieh, Christoph; Probst, Thomas; Felnhofer, Anna; Kothgassner, Oswald; Netzer, Ingeborg; Jesser,	Impact of the COVID-19 pandemic on the work of clinical psychologists in Austria: results of a mixed-methods study	2024	Frontiers in Psychology	10.3389/fpsyg.2024.1302442	https://www.frontiersin.org/articles/10.3389/fpsyg.2024.1302442/

Andrea					full
Sanwald, Alice; Theurl, Engelbert	Out-of-pocket expenditure by private households for dental services – empirical evidence from Austria	2016	Health Economics Review	10.1186/s13561-016-0087-5	https://healtheconomicsreview.biomedcentral.com/articles/10.1186/s13561-016-0087-5
Schaffler, Yvonne; Probst, Thomas; Jesser, Andrea; Humer, Elke; Pieh, Christoph; Stippl, Peter; Haid, Barbara; Schigl, Brigitte	Perceived Barriers and Facilitators to Psychotherapy Utilisation and How They Relate to Patient's Psychotherapeutic Goals	2022	Healthcare	10.3390/healthcare10112228	https://www.mdpi.com/2227-9032/10/11/2228
Schanda, H.; Stompe, T.; Ortwein-Swoboda, G.	Dangerous or merely 'difficult'? The new population of forensic mental hospitals	2009	European Psychiatry	10.1016/j.eurpsy.2009706	https://www.cambridge.org/core/product/identifier/S0924933800065639/type/journal_article
Schenk, Hannes; Heidinger, Petra; Insam, Heribert; Kreuzinger, Norbert; Markt, Rudolf; Nägele, Fabiana; Oberacher, Herbert; Scheffknecht, Christoph; Steinlechner, Martin; Vogl, Gunther; Wagner, Andreas Otto; Rauch, Wolfgang	Prediction of hospitalisations based on wastewater-based SARS-CoV-2 epidemiology	2023	Science of The Total Environment	10.1016/j.scitotenv.2023.162149	https://linkinghub.elsevier.com/retrieve/pii/S0048969723007659
Schuster, Raphael; Pokorny, Raffaela; Berger, Thomas; Topooco, Naira;	The Advantages and Disadvantages of Online and	2018	Journal of Medical Internet Research	10.2196/11007	https://www.jmir.org/2018/12/e

Laireiter, Anton-Rupert	Blended Therapy: Survey Study Amongst Licensed Psychotherapists in Austria				11007/
Burkert, Nathalie; Rásky, Éva; Freidl, Wolfgang	Social inequalities regarding health and health behaviour in Austrian adults	2012	Wiener klinische Wochenschrift	10.1007/s00508-012-0164-7	http://link.springer.com/10.1007/s00508-012-0164-7
Sögner, P.; Zur Nedden, D.	Stand der Telemedizin in Österreich	2005	Bundesgesundheitsblatt - Gesundheitsforschung - Gesundheitsschutz	10.1007/s00103-005-1055-0	http://link.springer.com/10.1007/s00103-005-1055-0
Spary-Kainz, Ulrike; Semlitsch, Thomas; Rundel, Sophie; Avian, Alexander; Herzog, Sereina; Jakse, Heidelinde; Siebenhofer, Andrea	How many women take oral supplementation in pregnancy in Austria?: Who recommended it? A cross-sectional study	2019	Wiener klinische Wochenschrift	10.1007/s00508-019-1502-9	http://link.springer.com/10.1007/s00508-019-1502-9
Stamm, Tanja; Ritschl, Valentin; Platzer, Alexander; Omara, Maisa; Mosor, Erika; Reichardt, Berthold; Schmitl, Lina; Behanova, Martina; Bekes, Katrin	Regional and gender differences in population-based oral health insurance data	2020	Clinical Oral Investigations	10.1007/s00784-019-03090-w	http://link.springer.com/10.1007/s00784-019-03090-w
Haririan, Hady; Pachel-Tettinger, Dagmara; Kirchner, Johannes; Moritz, Andreas; Rausch-Fan, Xiaohui	The evolution of treatment over 80 years at the outpatient department of the Viennese school of dentistry	2019	Community Dentistry and Oral Epidemiology	10.1111/cdoe.12424	https://onlinelibrary.wiley.com/doi/10.1111/cdoe.12424
Schneidtinger, Cornelia; Haslinger-Baumann, Elisabeth	The lived experience of adolescent users of mental health services in	2019	Journal of Child and Adolescent Psychiatric Nursing	10.1111/jcap.12245	https://onlinelibrary.wiley.com/doi/10.111

	Vienna, Austria: A qualitative study of personal recovery				1/jcap.12245
Urban, Matthias; Burghuber, Otto Chris; Dereci, Canan; Aydogan, Masite; Selimovic, Eldin; Catic, Selmir; Funk, Georg-Christian	Tobacco addiction and smoking cessation in Austrian migrants: a cross-sectional study	2015	BMJ Open	10.1136/bmjopen-2014-006510	https://bmjopen.bmj.com/lookup/doi/10.1136/bmjopen-2014-006510
Winkler-Pjrek, Edda; Spies, Marie; Baldinger-Melich, Pia; Perkmann, Lisa; Kasper, Siegfried; Winkler, Dietmar	Use of Light Therapy by Office-Based Physicians	2016	Neuropsychobiology	10.1159/000477094	https://karger.com/NPS/article/doi/10.1159/000477094
Van Lieshout, Jan; Frigola Capell, Eva; Ludt, Sabine; Grol, Richard; Wensing, Michel	What components of chronic care organisation relate to better primary care for coronary heart disease patients? An observational study	2012	BMJ Open	10.1136/bmjopen-2012-001344	https://bmjopen.bmj.com/lookup/doi/10.1136/bmjopen-2012-001344
Vonck, Kristl; Biraben, Arnaud; Bosak, Magdalena; Jennum, Poul Jørgen; Kimiskidis, Vasilios K; Marusic, Petr; Mitchell, James W.; Ferreira, Lara N.; Ondrušová, Martina; Pana, Adrian; Persson, Ulf; Von Oertzen, Tim J.; Lattanzi, Simona	Usage and impact of patient-reported outcomes in epilepsy	2023	Brain and Behavior	10.1002/brb3.3342	https://onlinelibrary.wiley.com/doi/10.1002/brb3.3342
Wagner, Gudrun; Zeiler, Michael; Waldherr, Karin; Philipp, Julia; Truttmann, Stefanie; Dür, Wolfgang; Treasure, Janet L.; Karwautz, Andreas F. K.	Mental health problems in Austrian adolescents: a nationwide, two-stage epidemiological study applying	2017	European Child & Adolescent Psychiatry	10.1007/s00787-017-0999-6	http://link.springer.com/10.1007/s00787-017-0999-6

	DSM-5 criteria				
Weiss, Gertraud; Steinacher, Ina; Lamprecht, Bernd; Kaiser, Bernhard; Mikes, Romana; Sator, Lea; Hartl, Sylvia; Wagner, Helga; Studnicka, M.	Development and validation of the Salzburg COPD-screening questionnaire (SCSQ): a questionnaire development and validation study	2017	npj Primary Care Respiratory Medicine	10.1038/s41533-016-0005-7	https://www.nature.com/articles/s41533-016-0005-7
Wieczorek, Christina C; Schmied, Hermann; Dorner, Thomas E; Dür, Wolfgang	The bumpy road to implementing the Baby-Friendly Hospital Initiative in Austria: a qualitative study	2015	International Breastfeeding Journal	10.1186/s13006-015-0030-0	https://internationalbreastfeedingjournal.biomedcentral.com/articles/10.1186/s13006-015-0030-0
Wieczorek, Christina C.; Marent, Benjamin; Dorner, Thomas E.; Dür, Wolfgang	The struggle for inter-professional teamwork and collaboration in maternity care: Austrian health professionals' perspectives on the implementation of the Baby-Friendly Hospital Initiative	2016	BMC Health Services Research	10.1186/s12913-016-1336-3	http://bmchealthservres.biomedcentral.com/articles/10.1186/s12913-016-1336-3
Wiedermann, Christian J.; Marino, Pasqualina; Van Der Zee-Neuen, Antje; Mastrobuono, Isabella; Mahlknecht, Angelika; Barbieri, Verena; Wildburger, Sonja; Fuchs, Julia; Capici, Alessandra; Piccoliori, Giuliano; Engl, Adolf; Østerås, Nina; Ritter, Markus	Patient-Reported Quality of Care for Osteoarthritis in General Practice in South Tyrol, Italy: Protocol for Translation, Validation and Assessment of the OsteoArthritis Quality Indicator Questionnaire	2023	Methods and Protocols	10.3390/mps6020028	https://www.mdpi.com/2409-9279/6/2/28

	(OA-QI)				
Winkler, Dietmar; Kaltenboeck, Alexander; Frey, Richard; Kasper, Siegfried; Pjrek, Edda	Changes over time of the diagnostic and therapeutic characteristics of patients of a psychiatric intensive care unit in Austria	2019	Comprehensive Psychiatry	10.1016/j.comppsych.2019604	https://linkinghub.elsevier.com/retrieve/pii/S0010440X19300276
Zechmeister-Koss, Ingrid; Winkler, Roman; Fritz, Corinna; Thun-Hohenstein, Leonhard; Tiichler, Heinz	Services Use of Children and Adolescents before Admission to Psychiatric Inpatient Care	nan	nan	nan	nan
Zechmeister-Koss, Ingrid; Aufhammer, Sandra; Bachler, Herbert; Bauer, Annette; Bechter, Philipp; Buchheim, Anna; Christiansen, Hanna; Fischer, Maria; Franz, Marianne; Fuchs, Martin; Goodyear, Melinda; Gruber, Nadja; Hofer, Alex; Hölzle, Laura; Juen, Evi; Papanthimou, Flora; Prokop, Mathias; Paul, Jean Lillian	Practices to support co-design processes: A case-study of co-designing a program for children with parents with a mental health problem in the Austrian region of Tyrol	2023	International Journal of Mental Health Nursing	10.1111/inm.13078	https://onlinelibrary.wiley.com/doi/10.1111/inm.13078
